# Evidence of a higher prevalence of HPV infection in HTLV-1-infected women compared to uninfected women

**DOI:** 10.1186/1742-4690-8-S1-A85

**Published:** 2011-06-06

**Authors:** Sônia Lopo, Paula Matos, Iuri Useda, Geisa Barbosa Pena, Maria Betânia Torrales, Viviana Olavarria, Rita E Mascarenhas, Bernardo Galvão-Castro, Maria F Rios Grassi

**Affiliations:** 1 Bahiana School of Medicine and Public Health (EBMSP) Salvador, Bahia, Brazil; 2 Advanced Laboratory of Public Health, Gonçalo Moniz Center, Fundação Oswaldo Cruz, Salvador, Bahia, Brazil

## Background

HTLV-1 increases susceptibility to infections. Few studies have addressed the co-infection between HPV/HTLV-1 and the immune response involved in this interaction.

## Aim

To determine prevalence of cervical HPV infection and to evaluate HTLV-proviral load and CD4 T lymphocyte proportion in co-infected patients.

## Methods

A cross-sectional study was carried out in Salvador-Brazil, between September 2005 to December 2008, involving 50 HTLV-1-infected women from the HTLV Reference Center and 40 uninfected patients from gynecological clinic, both at the Bahiana School of Medicine. HPV infection was assessed by hybrid capture. HTLV-1 proviral load was quantified by real time PCR and CD4+ T-lymphocyte count using flow cytometry.

## Results

Mean age of HTLV-1-infected women (38±10 yrs) was similar to that of the control group (36±13 yrs). The prevalence of HPV infection was 44% in the HTLV-1-infected group and 22.5% in uninfected women (p=0.03). HTLV-1-infected women had lower mean age at onset of sexual life (17±3 yrs vs. 19±3 yrs; p=0.03) and greater number of lifetime partners compared to the control group (4±3 vs. 2±1; p<0.01). In the group of HTLV-1-infected patients, there was neither difference in HTLV-1 proviral load between HPV-infected women (16,000 copies/10^6^ PBMC) and uninfected patients (6,114 copies/10^6^ PBMC, p=0,4) nor in the proportion of CD4+ T-lymphocytes (43.8±10.6% vs. 48.9±8.6%, p=0.1).

## Conclusion

The prevalence of HPV infection is higher in HTLV-1-infected women. No association was found between HPV infection, CD4+ T lymphocyte proportion or HTLV-1-proviral load in HTLV-1-infectred patients. Further studies should be performed to evaluate the progression of this co-infection.

